# Clinical Holding in Pediatric Venipuncture: Caring by Empowering the Caregiver

**DOI:** 10.3390/ijerph18147403

**Published:** 2021-07-11

**Authors:** Manuel Tomás-Jiménez, Elena Fernández Díaz, María Jesús Flores Sánchez, Andrea Navarro Pliego, Ramon Mir-Abellán

**Affiliations:** 1 Patient Safety Research Group, Hospital Parc Sanitari Sant Joan de Déu, Sant Boi de Llobregat, 08830 Barcelona, Spain; andrea.navarro@sjd.es (A.N.P.); ramon.mir@sjd.es (R.M.-A.); 2 Pediatric Service, Hospital Parc Sanitari Sant Joan de Déu, Sant Boi de Llobregat, 08830 Barcelona, Spain; elena.fernandez@sjd.es (E.F.D.); majesus.flores@sjd.es (M.J.F.S.)

**Keywords:** phlebotomy, children, adolescent, Clinical Holding, parent-child relations

## Abstract

The use of restraint in the child-adolescent population is highly controversial due to the consequences it can have for patients and their families, although it is sometimes necessary to employ restraint to perform techniques safely and effectively. Clinical Holding is committed to the involvement of parents during venipuncture in the context of family-centred care. This study assesses levels of distress and pain in children undergoing this procedure, as well as satisfaction in parents and nurses. Parents assist in the restraint of children and provide accompaniment during venipuncture. Levels of distress and pain were not particularly elevated. Satisfaction levels among parents and nurses were high. A positive correlation was found between anticipatory and real distress (r = 0.737, *p* = 0.000), and between real distress and real pain (r = 0.368, *p* = 0.035). A negative correlation was observed between real pain and parent satisfaction (r = −0.497, *p* = 0.003). Parental participation during venipuncture contributed to better management of distress and pain. In the future, it would be advisable to incorporate the other pharmacological and non-pharmacological measures recommended by Clinical Holding to ensure care of the highest quality and safety.

## 1. Introduction 

The use of restraint in children can lead to a series of physical consequences such as pain, a variety of injuries, or even speech disorders [1] and psychological repercussions that can range from emotional distress to ineffective coping skills and phobias that can complicate relationships with healthcare professionals [2]. There may even be negative effects on the emotional development of children, which result in physical and psychological problems in the future [3,4]. Apart from the risks involved for the child, restraints also have negative effects at the emotional and psychological level on the families and on the health professionals themselves [5].

Restraint of children should be considered a measure of last resort in the healthcare setting when other verbal and non-verbal techniques have failed, and with the aim of avoiding harm to the child, other patients or professionals. This represents a particular challenge in the area of physical health as many techniques require a certain level of restraint for them to be applied swiftly, effectively and without harming the patient. If such action is necessary, the period of application and the degree of force applied should be kept to a minimum [6,7].

Of all the medical procedures that children undergo, those involving needles are considered to be among the most feared by children [8,9,10,11]. It should be added that the fear resulting from a stressful situation can produce an increased perception of pain in patients [10,11,12]. Another factor to be taken into account is the traumatic effect on parents of witnessing their child being subjected to painful medical procedures [13,14].

Venipuncture is when a vein is pierced by a needle for either intravenous injection or the removal of blood, for diagnostic or therapeutic purposes [15]. It is estimated that children experience moderate-severe pain during venipuncture procedures [10,11] and it should be emphasised that if this pain is not adequately addressed, it can have negative effects on the child. These may involve anxiety and a heightened sensation of pain in future procedures, needle phobia, reduced adherence to treatments, a decrease in the analgesic effectiveness of treatments and avoidance behaviour with respect to medical devices [16,17].

In 2010, the Royal College of Nursing published a guide on the management of restrictive interventions in children and young people in which the term Therapeutic Holding was presented [6]. In 2019, the guide was updated and the new term Clinical Holding was introduced [18].

Clinical Holding is defined as immobilisation through the limited use of force in order to carry out a painful clinical procedure (venipuncture, lumbar puncture, etc.) quickly and effectively with the consent of the child. There are various synonyms for Clinical Holding including “Therapeutic Holding”, “Supportive Holding” and “Holding Still” [6,18,19,20,21]. The difference between Clinical Holding and Restrictive Physical Intervention (restraint) rests on the degree of force required, the patient’s consent and the underlying intent [5,6,18,19,22].

However, it is frequently difficult to determine at which point Clinical Holding ends and restraint begins, considering that the terms are opposite poles of the same continuum [5,19]. Jeffery (2010) suggests that the moment during the procedure when the child resists, does not consent or withdraws consent given previously, and that this leads to the application of more force than intended, it becomes restraint [19].

To put this into practice, the Royal College of Nursing, through Clinical Holding, supports the use of pharmacological (analgesia or sedation) and non-pharmacological (e.g., comfortable position, distractions and procedure information) techniques to manage anxiety and pain during clinical procedures. They give paramount importance to informing relatives and involving them wherever possible in any restraining process so that they move from being spectators to active participants during the procedure [6,18]. It is important to point out that the involvement of the parents in the procedure should be entirely voluntary and if the parents do not wish to remain present or to participate in restraint, the health professionals should not make them feel guilty [23]. 

Various authors support the presence and active participation of the parents during procedures as this has a calming effect on the children and makes the event less traumatic for them [24,25,26]. Nevertheless, it should be borne in mind, as reported by Melhuish and Payne, that the parents’ own anxiety can increase anxiety in the child and even heighten the perception of pain [27].

Family-centred care is defined as “a way of caring for children and their families in health facilities that ensures that this care covers the whole family, not only the child, and in which all family members are recognised as care recipients” [28]. A fundamental principle of family-centred care is the development of a trusting therapeutic relationship between the child, the parents and the health professionals. However, efforts have been made in the last decade internationally to decrease the use of health facilities and length of hospital stays in favour of greater use of outpatient services and day hospitals. This has led to a reduction in contact time between health professionals and the nuclear family, which complicates the establishment of this relationship [29].

The Royal College of Nursing and the National Institute for Health and Care Excellence (NICE) support the active involvement of parents during their children’s clinical procedures [6,7,18], in accordance with family care guidelines. Thus, the aims of this study were, first, to assess distress and pain in children and adolescents undergoing venipuncture and, second, to determine the degree of satisfaction of parents and nurses involved in the process.

## 2. Materials and Methods

### 2.1. Design

A cross-sectional correlational design without a control group was used in which consecutive, non-probabilistic sampling was conducted for participant recruitment.

### 2.2. Ethical Approval

This study was approved by the Clinical Research Ethics Committee at Fundació Sant Joan de Déu, with reference code number PIC-180-20. All participants received verbal and written information on the study and accepted participation voluntarily through provision of signed informed consent. 

### 2.3. Participants

The sample consisted of 33 children and adolescents attending hospitalisation and emergency services at a hospital in Barcelona province. Inclusion criteria were persons aged 4–14 years with a scheduled venipuncture procedure, either for intravenous injection (vessel cannulation) or blood removal. 

Those participants with deficits or psychomotor alterations that would complicate the assessment of the variables were excluded. Parents and relatives presenting linguistic barriers were also excluded. Patients with urgent or life-threatening venipuncture scheduled were excluded for clinical reasons. 

### 2.4. Variables and Instruments

#### 2.4.1. Main Variables

Anticipatory distress: distress manifested when the patient is informed about the venipuncture (1–5 min prior to the venipuncture); measured using the Groningen Distress Scale.

Real distress: distress manifested during venipuncture; measured using the Groningen Distress Scale.

Anticipatory pain: pain that the patient expects to receive once he or she is informed of the venipuncture (1–5 min prior to venipuncture); assessed through the Faces Pain Scale -Revised (FPS-R) scale in patients between 4 and 7 years, and the Numeric Scale in patients aged 8 years or older.

Real pain: pain manifested by the patient after venipuncture; assessed through the FPS-R scale in patients between 4 and 7 years, and the Numeric Scale in patients aged 8 years or older. 

Degree of family cooperation: degree of family collaboration during the procedure. Classified as grade 1 (cooperative, calms the child), grade 2 (nervous but attempts to calm the child), grade 3 (very nervous and does not calm the child) and grade 4 (intensely nervous, does not cooperate or calm the child). 

Family satisfaction: degree of satisfaction of family member on completion of the procedure; measured through the Net Promotor Scale (NPS).

Nurses’ satisfaction with the procedure: degree of nurses’ satisfaction on completion of the procedure; scored through a close-ended question with response options from 1 (Very dissatisfied) to 5 (Very satisfied).

#### 2.4.2. Demographic Data and Other Variables

Demographic variables related to the child’s age and sex were collected. Data were also gathered on the presence of prior negative experiences with venipuncture (Yes/No) and whether or not the child had previously been hospitalised (Yes/No).

In addition, information was recorded on the service, the technique (cannulation of the vessel/venipuncture), number of attempts until the procedure was successfully carried out, and requirements for extra personnel to hold the child.

### 2.5. Instruments

#### 2.5.1. Groningen Distress Scale

The Groningen Distress Scale is an observational scale developed by Humphrey et al. in 1992 [30] and designed to measure anxiety during a brief medical procedure. It is a scale based on the Behavioural Approach-Avoidance and Distress Scale (BAADS) and in the original article this scale was used to measure anxiety levels during venipuncture in a child-adolescent population aged between 2½ and 19 years.

The scale rates distress or anxiety in the child on 5 levels from low to high (1–5). Crying and muscle tension, both of which are relatively easy to define operationally, are the two most frequently presented behaviours and thus are the two main variables taken into account. Distress is classified into 5 degrees: 1- Calm, 2- Shy/nervous, 3- Moderate anxiety but still under control, 4- Continuous tension and crying, 5- Panic.

#### 2.5.2. Faces Pain Scale Revised (FPS-R)

FPS-R is a self-assessment scale created by Hicks et al. [31] and widely-used in the pediatric health setting [32] to evaluate pain through a series of faces that progressively express distinct degrees of pain. Its use is indicated to assess procedural and post-surgical pain in children aged 4 years and older. It is straightforward to apply; the child selects the face that most closely corresponds to the pain they feel. Words such as “happy” or “sad” should be avoided while the child is performing the self-assessment so that he or she focuses solely on the pain and bias is not introduced into the result. It consists of 6 faces, scored from left to right as 0, 2, 4, 6, 8 and 10, where 0 is “No pain” and 10 is “A lot of pain”.

#### 2.5.3. Numeric Pain Scale

Self-assessment scale created by McCaffery, Beebe et al. [33,34] to evaluate pain and indicated for procedural and post-surgical pain in children aged 8 years and older. It consists of a line with numbers from 1–10 on which the child points to the number corresponding to the pain he or she feels or simply says the number.

Both pain scales are integrated into the same visual analogue scale and the numeric equivalence of pain oscillates between None (0), Mild (1–3), Moderate (4–6) and Severe (7–10).

#### 2.5.4. Net Promoter Scale (NPS)

The NPS was created in 1993 by Fred Reichfield and while it was originally used in the field of finance [35], it is also currently used in the health setting. This system measures the degree of satisfaction among clients with a simple question: “How likely are you to recommend the company to a relative or friend?” The client then selects a score from 0 to 10 where 0 represents the least likelihood and 10 the greatest likelihood.

According to the score obtained, the clients are classified into three categories: Promotors (score 9–10), Neutrals (score 7–8) and Detractors (score 0–6). 

The final calculation depends on subtracting the percentage of detractors from the percentage of promotors, ignoring the scores of the neutrals. The final indicator oscillates between −100 and +100, where a score over 50 points is considered an indicator of a good experience on the part of the client.

### 2.6. Procedure

The children and their families who were attended to at the hospital were invited to participate voluntarily through an information sheet and then provided signed informed consent.

The child and its family were informed by the nurse about the scheduled venipuncture technique, highlighting the holding and accompanying role of the family during the procedure and answering any queries that may arise. Subsequently, after obtaining the child’s consent, a health professional performed the anticipatory distress and pain assessment before proceeding with the venipuncture. The child was placed on the bed in a supine position and the venipuncture was performed. The parents accompanied the child and cooperated with a gentle hug while reassuring the child. As the nurse performed the venipuncture, another staff member held the limb in the correct position to ensure the success of the technique. 

During venipuncture, the degree of cooperation received from the family was assessed along with the real distress presented by the child. On completion of the technique, the real pain expressed by the child and the degree of parent satisfaction were assessed and the nurse self-assessed his or her degree of satisfaction with the procedure. Following the process, patients were given bravery stickers as positive reinforcement.

### 2.7. Data Analysis

All data analyses were carried out using SPSS version 21.0 (IBM Corporation, Armond, NY, USA). 

Quantitative variables were expressed as mean, SD, Min and Max. 

Pearson or Spearman’s Correlation Coefficient were used for the comparison between quantitative variables depending on the normality of distribution. Categorical variables were expressed as frequencies and percentages. The Chi-squared test was used for the comparison between groups and categorical variables. 

The Shapiro–Wilk test was used to assess the normality of the samples, due to the sample size (*n* = 33). 

Statistical significance was set at *p* < 0.05 (two tailed).

## 3. Results

### 3.1. Socio-Demographic Variables

The sample consisted of 33 patients of whom 54.5% (18) were male and 45.5% (15) were female. Overall, the mean age was 10.82 years (SD: 3.07), with 10.56 years (SD: 3.11) for males and 11.13 years (SD: 3.11) for females. 

### 3.2. Distress Analysis

Total anticipatory distress in the sample had a mean of 1.97 (SD 0.95) while real distress was 1.91 (SD 0.94). Anticipatory distress was higher in females (M: 2 SD: 0.926) than in males (M: 1.94 SD: 0.998). In contrast, real distress was higher in males (M: 1.94 SD: 0.938) than in females (M: 1.87 SD: 0.990). Results were not statistically significant in either anticipatory distress (t: −0.164; p.: 0.762) or real distress (t: 0.231; p.: 0.698). 

Table 1 and Table 2 show the correlation between anticipatory and real distress and other variables of interest.

As can be observed in the tables above, there is a positive correlation between anticipatory distress and real distress with statistical significance. Results also show a statistically significant correlation between real distress and real pain. No statistically significant correlations were found between anticipatory and real distress and other variables. 

### 3.3. Pain Analysis

Anticipatory pain showed a mean of 3.39 SD: 3.36 while real pain had a mean of 2.76 SD: 2.894. In both cases minimum and maximum scores were 0 and 10, respectively, with the greatest percentage for the score of 0 in anticipatory pain (36.4%) and real pain (33.3%). 

Females showed an appreciably higher degree of anticipatory pain (M: 3.93 SD: 3.693) than males (M: 2.94 SD: 3.096), although this was not statistically significant (p: 0.409). Females also showed higher scores for real pain (M: 2.93 SD: 2.492) than males (M: 2.61 SD: 3.256) although this did not reach statistical significance.

Higher scores were observed for anticipatory pain related to the vessel cannulation technique (M: 4.38 SD: 3.321) than for venipuncture (M: 0.78 SD: 1.716), with a statistically significant association (p: 0.004).

Table 3 and Table 4 show the correlation coefficients for anticipatory and real pain, respectively.

In anticipatory pain, no statistically significant correlations were found with the variables shown in Table 3. In the case of real pain, a statistically significant positive correlation was shown between real pain and real distress (Table 2 and Table 4). A statistically significant strong negative correlation was also seen between real pain and parent satisfaction. 

### 3.4. Parents’ Degree of Cooperation and Satisfaction

Regarding parents’ degree of cooperation, 90.9% of parents were categorised as “Grade 1: Cooperative, calms the child” and only 9.1% were categorised as “Grade 2: Nervous but attempts to calm the child”. 

Parent satisfaction showed a mean of 9.79 SD: 0.485, with 3% scoring 8, 15.2% scoring 9 and 81.8% scoring 10. Parents showed a high degree of satisfaction with the process. Parent satisfaction did not show a correlation with any of the variables except the negative correlation with real pain (r: −0.497, *p*: 0.003) and the positive correlation approaching statistical significance with nurse satisfaction (r: 0.335, *p*: 0.057).

To calculate the global score on the parent satisfaction NPS scale, the neutrals are ignored (scores of 7–8), and since there are no detractors, the global score is +100, which indicates an excellent experience on the part of the parents. 

### 3.5. Nurse Satisfaction

Nurse satisfaction had a mean of 4.758 SD: 0.501, with 3% categorised as “Neither satisfied nor dissatisfied”, 18.2% as “Satisfied” and 78.8% as “Very satisfied”, showing a high degree of satisfaction among nurses with the process. Nurse satisfaction did not show a correlation with any of the variables, although positive correlation values approaching statistical significance were observed with parent satisfaction as mentioned above. 

### 3.6. Other Variables

No relationships were found with previous hospitalisations or previous negative experiences and the other study variables.

## 4. Discussion

The aims of this study were to evaluate distress and pain in children and adolescents undergoing venipuncture and determine the degree of satisfaction of parents and nurses involved in the process. Despite the size of the study sample (*n* = 33), the participation of parents during the venipuncture procedure and the holding and calming of the child revealed interesting results.

Levels of anticipatory and real distress detected were not very high, although an association was found between anticipatory and real distress and this finding is in line with those of other studies that examined the relationship [36,37]. An association was also observed between real distress and real pain manifested by the patient, which underlines the direct influence that distress can have on perceived pain in a patient exposed to a stressful situation, such as undergoing a venipuncture procedure [10,11,12].

Some authors point out that the position during venipuncture can be critical in the management of distress. The supine position, as used in this study, is associated with intensification of the feelings of fear and loss of control and this may contribute to more acute anticipation of the pain that will be felt [38]. Some researchers have studied the influence of the upright position with the parents cooperating by holding the child in a gentle hug and have reported reduced distress, less pain and greater satisfaction on the part of the parents and the child [39,40,41]. 

The study by Cavender did not find statistically significant differences when comparing the supine and upright positions, although the trend, as indicated by the size of the sample, favoured the upright posture [42]. Taddio et al. concluded that an affectionate embrace from the parents was welcomed by the child and had a reassuring effect that increased the feeling of safety and comfort while helping to keep the child’s body and limb still in preparation for the venipuncture, thus avoiding the need for any more staff involvement than was absolutely necessary [17].

Concerning anticipatory and real pain, the figures were not high, with the mean for anticipatory pain categorised as moderate and real pain mean as mild. Other authors have reported that venipuncture related pain is rated by pediatric patients as moderate-severe [10,11], making it one of the most feared procedures in this group. Results reveal that the highest degree of anticipatory pain is associated with vessel cannulation, which may be connected to the belief among children that the needle remains in the vein. However, the perception of real pain during vessel cannulation was lower than anticipated. The decrease in the perception of real pain in the present study could be associated with the calming effect of the parents. 

No relationship was found between distress or pain and age, which is in contrast to the findings of previous studies in which younger ages were related to greater degrees of pain and anxiety manifested during venipuncture [30,43]. 

In the sample under study, all parents cooperated during the procedure by accompanying, calming the child and gentle holding in a suitable manner in the supine posture. This high degree of cooperation is certainly due to the detailed information and accompaniment provided to the families by the nurse, which takes into account the entire nuclear family rather than just the patient in question. The high degree of satisfaction on the part of the nurse and its relationship with parent satisfaction could indicate the professionals’ acceptance of parents as participating agents during the clinical procedure. This finding is in accordance with those of other studies that positively rate the calming presence and participation of parents during the process [24,25,26]. 

Nevertheless, the negative relationship observed in the results between parent satisfaction and real pain expressed by children should be highlighted. The greater the pain manifested by the child, the lower the satisfaction of the parents. This is particularly relevant given the low levels of pain found and the high parent satisfaction scores, which reinforce the need to employ pharmacological and non-pharmacological measures for the management of pain during venipuncture.

There is a certain controversy regarding the experience of previous venipuncture procedures and manifested stress, as there are some authors who report that children without previous experience manifest lower stress than those with experience [37,43], while other authors found no relationship between previous experience and anxiety [44]. In this study we did not find any relationship between previous negative experiences or previous hospitalisations and levels of anxiety and pain. 

### Implications of the Study

Some authors support the use of pharmacological measures such as topical anesthetic [45,46] and non-pharmacological measures such as distraction for the management of pain during painful procedures where the patient can choose their preferred method [47]. Cognitive-behavioural strategies including orientation through images or stories are also a useful tool that can offer information to the patient on what is about to take place in a manner adapted to their understanding to reduce anxiety regarding the unknown [48]. The incorporation of these methods can contribute to improving the distress and pain scores obtained.

The inclusion of parents during the procedures can contribute to improving the experience for child patients, as well as strengthening the alliance between health personnel and families. 

Clinical Holding offers a series of pharmacological and non-pharmacological measures that ensure the highest quality care and safety that can be applied to a wide range of techniques usually carried out in clinical practice to enhance the experience of both patients and families. 

## 5. Conclusions

The present study shows the importance of involving family members as they can contribute to the management of distress and perceived pain in a technique that is as frequent and feared as venipuncture. Results reveal high parent satisfaction at being active participants in the care of their children and directly cooperating with health professionals. The limitations of this study are linked to the size of the sample; thus it would be advisable to replicate the study using larger samples to compare results. A further limitation was the absence of a control group, which prevented direct associations being described between results and the intervention itself, although, for ethical reasons, a decision was taken not to deprive any child of the presence of his/her parents during the procedure. Another limitation that should also be mentioned is the low pain scores observed, taking into account that the sample was not very distressed. We should also point out that the age range (4–14 years) is very wide and, as such, the sample contains various developmental abilities, coping skills and reliance on parental support. This wide age range may also account for the low pain scores observed in the sample. 

The best way to care for caregivers is to empower them and this empowerment is achieved by involving caregivers in the health processes of the people they look after, with the accompaniment and support of health professionals throughout.

Future research should use larger sample sizes and a narrower age range to better represent the population group under study. Collection of sociodemographic data on parents accompanying children during the procedure should also be considered. In addition, future studies should use the whole bundle of activities recommended by Clinical Holding (pharmacological and non-pharmacological measures), and consider transferring these to other procedures carried out in child-adolescent care. 

## Figures and Tables

**Table 1:** Spearman’s correlation coefficient. Anticipatory distress.

Variable	r	*p*
Age	−0.019	0.916
Real Distress	0.737	0.000 S
Anticipatory Pain	0.241	0.231
Real Pain	0.157	0.384
Parent Satisfaction	−0.166	0.357
S: Significant		

**Table 2:** Spearman’s correlation coefficient. Real distress.

Variable	r	*p*
Age	0.104	0.564
Anticipatory Distress	0.737	0.000 S
Anticipatory Pain	0.138	0.444
Real Pain	0.368	0.035 S
Parent Satisfaction	−0.195	0.276
S: Significant		

**Table 3:** Spearman’s correlation coefficient. Anticipatory pain.

Variable	r	*p*
Age	−0.116	0.522
Anticipatory Distress	0.214	0.231
Real Distress	0.138	0.444
Real Pain	0.249	0.162
Parent Satisfaction	−0.296	0.094
S: Significant		

**Table 4:** Spearman’s correlation coefficient. Real pain.

Variable	r	*p*
Age	−0.243	0.173
Anticipatory Distress	0.157	0.384
Real Distress	0.368	0.035 S
Anticipatory Pain	0.249	0.162
Parent Satisfaction	−0.497	0.003 S
S: Significant		

## Data Availability

The data presented in this study are available upon request from the corresponding author.
